# High levels of isotope elimination improve precision and allow individual-based measurements of metabolic rates in animals using the doubly labeled water method

**DOI:** 10.14814/phy2.12552

**Published:** 2015-11-26

**Authors:** Masaki Shirai, Yasuaki Niizuma, Maki Yamamoto, Emiko Oda, Naoyuki Ebine, Nariko Oka, Ken Yoda

**Affiliations:** 1 Graduate School of Environmental Studies, Nagoya UniversityNagoya, Japan; 2 Faculty of Agriculture, Meijo UniversityTenpaku-ku, Nagoya, Japan; 3 Department of Bioengineering, Nagaoka University of TechnologyNagaoka, Niigata, Japan; 4 Faculty of Health and Sports Science, Doshisha UniversityKyotanabe, Kyoto, Japan; 5 Division of Natural History, Yamashina Institute for OrnithologyAbiko, Chiba, Japan

**Keywords:** Doubly labeled water method, respirometry, seabird, validation

## Abstract

Doubly labeled water (DLW) can be used to measure energy expenditure in free-ranging animals, but questions have been raised about its accuracy in different species or contexts. We investigated whether differences in the extent of isotope elimination affects the precision and accuracy of the DLW method, which can vary according to the experimental design or metabolic rate of the species. Estimated total energy expenditure by the DLW method (TEE_dlw_) was compared with actual total energy expenditure simultaneously measured via respirometry (TEE_resp_) in streaked shearwaters *Calonectris leucomelas*, a pelagic seabird. Subjects were divided into three groups with different experimental conditions: at rest on the ground for 24 h (Group A) or for 48 h (Group B), and at rest on the water for 24 h (Group C). TEE_dlw_ in Group A matched TEE_resp_, whereas there was an overestimation of TEE_dlw_ in both Groups B and C compared with TEE_resp_. However, compared with Group A, TEE_dlw_ in Groups B and C had reduced the isotopic analytical variability and thus higher precision. The best regression model (TEE_dlw_ = 1.37 TEE_resp_ − 14.12) showed a high correlation (*R*^2^ = 0.82) between TEE_dlw_ and TEE_resp_ and allows a correction factor for field metabolic rates in streaked shearwaters. Our results demonstrate that the commonly made assumption that the DLW method is not appropriate for individual-based estimates may be incorrect in certain circumstances. Although a correction factor may be necessary when using the DLW method to estimate metabolic rate, greater levels of isotope eliminations provides DLW estimates with high precision, which can adequately represent relative individual estimates. Nevertheless, the DLW method, should be used with caution when characterizing interspecies difference of energy expenditures.

## Introduction

The balance between energy acquisition and energy expenditure strongly impacts survival and reproductive success of animals (Kitaysky et al. 2000; Ricklefs and Wikelski 2002; Golet et al. 2004). Individual variation in field metabolic rate (FMR) is often large (e.g., Fyhn et al. 2001; Welcker et al. 2010) and may influence reproductive performance (e.g., Wendeln and Becker 1999), so an individual’s ability to adjust energy expenditure might, therefore, be a good predictor of fitness (Drent and Daan 1980). Even so, limited studies have investigated the proximate factors of individual variation of energy expenditures in wild animals (Bryant and Tatner 1991; Tinbergen and Dietz 1994; Peterson et al. 1998), mainly because of the limitations of the methodologies available for measuring energy expenditure in free-living animals (Butler et al. 2004).

To date, several methods have been used for measuring metabolism and energy expenditure in animals (Halsey 2011). Although each method includes both random and systematic error, the methods differ in accuracy (i.e., the closeness of an estimated value to its true value) and precision (i.e., the closeness of repeated measurements of the same quantity to each other). Respirometry, which is among the most commonly used techniques for measuring baseline energy metabolism – basal, standard, and resting metabolic rate (BMR, SMR, and RMR, respectively), has been used for at least 200 years (Halsey 2011). The resulting estimation of energy demands from an open-flow respirometric system, one of the main respirometry techniques, has high precision (coefficient of variation within 3%; Sparling et al. 2008) and accuracy (mean error within 3%; Withers 1977). However, respirometry systems require keeping the animal in a metabolic chamber and it is not possible to replicate the natural environment of the animal in the small chamber. Other methods have since been developed to measure metabolic rates of free-ranging animals (i.e., field metabolic rate) (Halsey 2011).

**Figure 1:**
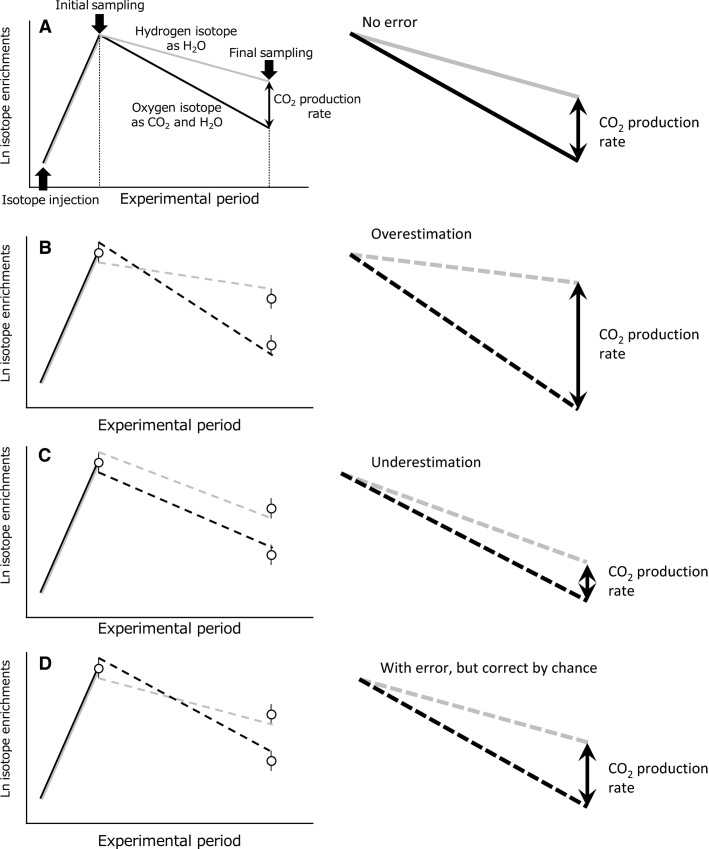
Schematic diagram of the causes of random error through isotopic analytical variability in the doubly labeled water method. (A) Isotopes flood the body water leading to a sharp exponential increase in enrichment until equilibrium is reached. Following the equilibrium, the isotopes are eliminated down exponential routes back to the background levels. Because oxygen isotope (black line) is eliminated in both H_2_O and CO_2_, its enrichment declines faster than that of hydrogen isotope (gray line), which is eliminated only in H_2_O. The difference in the isotope elimination rates provides a quantitative estimate of the rates of CO_2_ production. (B–D) Each isotope enrichment (open circle) of initial and final isotopes receives analytical variability in isotope ratio mass spectrometry (vertical solid line stuck on the open circle). The isotopic analytical variability causes a discrepancy between the actual and estimated CO_2_ production rates, which are provided from difference in the uncertain isotope elimination rates (black and gray dashed lines). Estimated CO_2_ production rates were largely (B) overestimated or (C) underestimated by the inverse analytical errors of initial and final sample. (D) However, because CO_2_ production rates were computed by the “rate” of isotope eliminations, there is little error on estimated CO_2_ production when the directions of the analytical errors correspond. Therefore, the propagations of analytical errors influence randomly estimated CO_2_ production rates.

Among the methods to measure FMR, the doubly labeled water (DLW) method is the most common technique for measuring animal energetics in the field. The DLW method estimates the rate of CO_2_ production (rCO_2_), which reflects metabolic rate of subjects (Lifson and McClintock 1966; Speakman 1997), and has been used to measure FMR of a wide range of free-living animals (Nagy et al. 1999; Ellis and Gabrielsen 2002; Speakman and Król 2010). When water labeled with stable isotopes of oxygen and hydrogen (^18^O and ^2^H) is injected into a subject, the isotopes are eliminated, mainly as CO_2_ and H_2_O. Since ^2^H leaves the animal via H_2_O, whereas ^18^O leaves via both CO_2_ and H_2_O, it is possible to estimate rCO_2_, from the difference in elimination constants, which can then be used to calculate to metabolic rate (Fig.1A). The method, however, has been believed to be too imprecise to estimate the energy expenditure of an individual subject (Butler et al. 2004) due to considerable random error generated through analytical variability (Nagy 1983). Since both the initial and final sample add analytical variability in isotope ratio mass-spectrometry (IRMS), the variability propagates and influence randomly calculated elimination rates and rCO_2_ (Fig.1B–D). Thus, the DLW method has been mainly limited to provide a mean estimated metabolic rate of a group of individuals, which would have a mean error within about 2–3% (Speakman 1997; Butler et al. 2004), although some studies have used the DLW method for a single individual (e.g., Harding et al. 2009; Scantlebury et al. 2014).

Nevertheless, there are some attempts to examine the relative contribution of intra- and interindividual differences on FMR measured via the DLW method (Speakman et al. 1994; Berteaux et al. 1996; Williams et al. 2009; Elliott et al. 2014). Some field studies using the DLW method, particularly studies of pelagic seabirds, show correlations between individual FMR and behavior or with environmental variables. For example, FMR in thick-billed murres *Uria lomvia* measured by the DLW method is strongly positively correlated with locomotion intensity measured by a miniature accelerometer (Elliott et al. 2013). In wandering albatrosses *Diomedea exulans*, FMR increases with an increase in the number of landings (Shaffer et al. 2001). Unsuitable wind conditions for flight also increase FMR in some seabirds (Furness and Bryant 1996; Mullers et al. 2009). A computer simulation study of the precision of the DLW method was conducted using an artificial FMR dataset of snakes with a randomly generated error between −20% and +20% (Appendix in Peterson et al. 1998). The result suggests that despite a possible discrepancy of individual FMR measurements of up to 20%, individual FMR correlates with other ecological variables (Peterson et al. 1998). This evidence suggests that FMR measured by the DLW method may have sufficient precision to detect correlations between energy expenditure and individual activity or with environmental variables (Shaffer 2011).

Since DLW measurements in the field are conducted using individuals performing energetically costly activities over the course of longer experimental periods, one might expect that subjects in field studies eliminate much larger quantities of isotope than subjects in validation studies. In fact, in DLW experiments, a high level of isotope eliminations is recommended at least until isotope levels of a subject become close to natural isotope abundance, because increased isotope eliminations are expected to make the isotopic analytical variability (i.e., random error) smaller and should therefore generate more reliable isotope elimination rates (Nagy 1983). The validation study in California sea lions *Zalophus californianus*, which resulted in isotope depletions of 9.0% in ^2^H and 13.8% in ^18^O, produced a mean coefficient of variation (%CV) of 35% in DLW estimates (Boyd et al. 1995), whereas a study in gray seals *Halichoerus grypus* accompanied with isotope depletions of 38% in ^2^H and 46% in ^18^O produced a mean %CV of 7% (Sparling et al. 2008). Metabolic rates in a poultry chick with isotope depletions of 73.0% in ^18^O (mean absolute errors [i.e., precision]: 3.9–6.9%) was more precise than that accompanied with isotope depletions of only 30% in ^18^O (precision: 10.5–17.0%) (Gessaman et al. 2004). In addition, metabolic rates in little penguins *Eudyptula minor* estimated from the DLW method were 10.9% higher than those estimated from a material balance after 2 days (isotope depletions of 28.1% in ^18^O), but only 1.7% higher after 6 days (isotope depletions of 70.3% in ^18^O) (Gales 1989). Although these observations imply that improved precision and/or accuracy of the DLW method by high isotope eliminations may allow reliable measurements of individual differences by the DLW method in the field, few validation studies have investigated the actual precision and accuracy on the DLW method considering situations in the field.

To investigate the precision and accuracy of the DLW method under an environment where a subject experiences different levels of isotope eliminations, we measured metabolic rate in streaked shearwaters *Calonectris leucomelas* using the DLW method, simultaneously with the respiromety during experimental periods that differed in length or in conditions affecting rates of energy expenditure: both expected to affect isotope elimination. In seabirds, metabolic rates while resting on the water is expected to be two or three times compared to when they are just resting on the ground (Bevan et al. 1995a; Richman and Lovvorn 2011). Thus, we compared metabolic rate among experimental birds measured over the course of 24 versus 48 h, and we compared metabolic rates in birds measured while resting on the ground versus resting on the water. During these tests, we compared metabolic rates estimated by the DLW method with actual metabolic rates concomitantly measured using the respirometric system, and we examined how the amount of eliminated isotope affects the precision and accuracy of metabolic rate measured by the DLW method.

## Materials and Methods

### Study site and species

Our study was conducted between August and October in 2010, 2012, and 2013 on Awashima Island (38°27′N, 139°13′E, Niigata, Japan), which is an inhabited island located in the Sea of Japan. More than 10,000 streaked shearwaters breed on the island (Oka 2004). Streaked shearwaters, which are a burrowing procellariiform seabird, breed on offshore islands of East and Southeast Asia and migrate to wintering areas in the tropics (Oka 2004; Yamamoto et al. 2010). They forage for pelagic fish and return to their colony only at night to feed a single chick (Ochi et al. 2010; Matsumoto et al. 2012). As with other shearwaters, their RMR is low compared to other seabirds due to their lower body temperatures (Warham 1990, 1996; Shirai et al. 2012b).

We caught 24 adult shearwaters (10 birds in 2010, five birds in 2012, and nine birds in 2013) at night in their burrows to obtain their metabolic rates measured by both the respirometry and DLW method. We divided the birds into three groups with different experimental conditions for producing different amount of isotope elimination: Group A, a measurement at rest on the ground for 24 h (10 birds); Group B, a measurement at rest on the ground for 48 h (five birds); and Group C, a measurement at rest on the water for 24 h (nine birds). Each bird was held in dark boxes, transported to the laboratory within 10 min, and kept for at least 2 h to minimize the effects of capture stress on the metabolic rate. After the experiment, the birds were immediately released back into their burrows and given a supplementary feeding of approximately 20 g of Japanese jack mackerel *Trachurus japonicus*. We also captured 22 other adult birds not used in the respirometry and DLW experiment (four birds in 2010 and nine birds in 2012 and 2013) and took 1 mL of blood to determine the natural background isotope abundances in each year. This work was conducted with permits from the Ministry of the Environment, and all experiments were performed according to a protocol approved by the Institutional Animal Care and Use Committee of Nagoya University.

### Respirometric method

Oxygen consumption rate (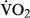) during the entire 24-h or 48-h period was measured using an open-flow respirometry system consisting of an acrylic metabolic chamber and an oxygen analyzer (Xentra 4100; Servomex, Crowborough, UK; Shirai et al. 2012a). For the measurement of metabolic rate on the ground, a 20-L metabolic chamber (20 cm long × 25 cm high × 40 cm wide) was submerged in a thermostatic water bath and maintained at 20.8 ± 0.8°C (mean ± SD). Measurements of metabolic rate while birds were on the water were obtained by filling a 72-L metabolic chamber (30 cm long × 60 cm high × 40 cm wide) with freshwater to a depth of 30 cm while the temperature of the water was maintained at 21.4 ± 1.7°C. Absorption of oxygen by water in the chamber was assumed to be negligible (less than 0.0015% per minute according to Allers and Culik 1997). The chamber temperature (*T*_c_) and atmospheric pressure (*P*_a_) were recorded using loggers (*T*_c_: ±0.7°C, Thermochron Type-SL; KN Laboratories, Ibaraki City, Osaka, Japan; *P*_a_: ±1.5 hPa, TR-73U Thermo Recorder; T&D Corp., Matsumoto City, Nagano, Japan) every minute. The flow rate through the chamber was maintained at 2.0 L/min (on the ground) and 5.0 L/min (on the water) using a mass flow controller calibrated by the manufacturer using hydrogen gas with a controlled flow rate (±2%, Type HM1171A; Tokyo Keiso, Minato City, Tokyo, Japan). The effluent air was dried over a water separator (AMG150C, SMC Co., Tokyo, Japan) and silica gel, which are connected in series, and a fraction of the dry effluent air was directed into the oxygen analyzer. The oxygen analyzer was calibrated using dry outside air (set to 20.946% oxygen) and pure stock nitrogen (set to 0.000% oxygen). The oxygen concentration in the effluent air was read by a computer every minute. 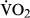 was calculated using formula 3A presented by Withers (1977). We assumed that respiratory exchange ratio (RER) = 0.8, which minimizes error in the estimated rate of energy expenditure when RER is unknown (Koteja 1996), and that the oxygen concentration of influent air was 20.946%. If RER = 0.8 is assumed, the error of estimating the rate of oxygen consumption is between −2.6% and +4.4% when the actual RER is between 0.7 and 1.0 (Koteja 1996). Initial (BM_i_) and final body mass (BM_f_) was measured using a spring scale (Pesola, Baar, Switzerland) with a scale division of 10 g and estimated to the nearest g before and after the bird was placed in the respirometric chamber, respectively. The body mass was assumed to decrease linearly from BM_i_ to BM_f_. A conversion coefficient of 20.1 kJ/L was used in calculating the energy expenditure from 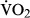 (RER = 0.8; Gessaman and Nagy 1988). Each bird’s energy expenditure was recorded every minute and these values were used to calculate the total energy expenditure for the whole duration of the experiment to allow comparison with energy expenditures measured using the DLW method.

For comparison with previous published data, we also calculated mass-specific resting metabolic rates (RMR) on the ground and on the water. Although previous publications used the term of BMR, RMR, or SMR to describe the baseline of energy metabolism, our study treat all these measures of resting metabolism as equivalent, and use RMR to represent resting metabolism. Since metabolic rate, even during resting, may be affected by slight changes in body temperature, hormone levels, and a host of other underlying physiological processes, shorter calculation intervals may lead to high stochastic variance (Hayes et al. 1992). On the other hand, longer calculation intervals may include periods of activity (Hayes et al. 1992). Thus, we calculated the minimal metabolic rate of the shearwater with a 30-min interval. All results are given at standard temperature and pressure for dry gas (STPD).

### Procedure for doubly labeled water method

Each shearwater was injected intraperitoneally with 2.5–3.0 g of DLW containing 10.2–12.2 atom-percent ^18^O (Taiyo Nippon Sanso, Shinagawa City, Tokyo, Japan), 5.5–5.8 atom-percent ^2^H (Isotech, Miamisburg, OH, USA), and 0.9% NaCl. To quantify the injected dose, the syringe was weighed before and after injection on an electrical balance (Mettler-Toledo, Columbus, OH) to the nearest 0.1 mg. After the injection, the bird was placed into a plastic box for 160–180 min to allow the injected dose to equilibrate. Then, 1 mL of blood was taken from the brachial or tarsal vein of the bird (initial sample), and the bird was placed in a respirometric chamber. To reduce the error caused by circadian metabolic rhythm, measurement period was adopted as a multiple of 24 h (Speakman and Racey 1988). Twenty-four hours or 48 h after taking initial samples, the bird was removed from the chamber, and 1 mL of blood was sampled from the brachial or tarsal vein (final sample).

Each blood sample was put into a heparinized tube and centrifuged immediately (6200 rpm for 5 min). The serum was then transferred to a plastic screw-cap vial with O-rings (AGC Techno Glass, Funabashi City, Chiba, Japan) and frozen at −25°C until isotope ratio analysis.

### Isotope ratio analysis

The hydrogen and oxygen isotope concentrations of the serum and DLW dose samples were analyzed according to the procedure of Shirai et al. (2012a) using isotope ratio mass spectrometry (IRMS; Hydra 20-20, Sercon, Crewe, UK; Yamada et al. 2009). The dose and serum samples were diluted with distilled water measured with an electrical balance (Mettler-Toledo, Columbus, OH) to the nearest 0.01 mg (Shirai et al. 2012a). The enrichment of distilled water was measured using IRMS, as with the diluted serum and dose samples.

The distilled water, diluted serum, and dose samples were put into a cylindrical tube and analyzed using the water equilibration method (Horita et al. 1989). Water standards (Iso-Analytical, Crewe, UK) were used to establish calibration curves for normalizing the values. Each sample was analyzed in duplicate. All isotope enrichments were measured in *δ* per mille relative to the working standards and converted to an absolute ratio for hydrogen isotope by using equation 14.4, and for oxygen isotope by using equation 14.9 from Speakman (1997). Absolute ratios were converted to ppm for hydrogen isotope by using equation 14.8, and for oxygen isotope equation 14.14 from Speakman (1997). All subsequent calculations in the DLW method were performed on the mean values of each sample analyzed in duplicate.

### Calculations in the DLW method

The plateau method was used to determine the isotope dilution spaces for hydrogen (*N*_d_, mol) and oxygen isotopes (*N*_o_, mol), and to estimate total body water pool (TBW) (Speakman 1997; Jacobs et al. 2012). For the calculations of rCO_2_, the dilution space ratio (*R*_dilspace_) was also obtained as *N*_d_/*N*_o_ (Speakman 1997). The elimination rates for hydrogen and oxygen isotopes (*k*_d_ and *k*_*o*_, respectively, per day) were determined using the two-sample technique (Lifson and McClintock 1966; Speakman 1997).

Ideally, background isotope levels should be determined for each animal before injection with labeled water (Speakman and Racey 1987). However, this increases both the handling time and disturbance to the animal. Thus, we determined the natural background isotope abundances in 22 uninjected adult shearwaters. We used the mean background levels of each year to calculate the CO_2_ production rate (rCO_2_, mL/day) (see Table S2).

rCO_2_ was calculated for each trial using several different published models with different assumptions about evaporative water loss and different combinations of body water pool estimates. The calculation models are largely categorized into two types based on different assumed body temperatures (25°C or 37°C; Speakman 1997). We used the following five equations including isotope fractionation factors measured at 37°C, because an assumed body temperature of 37°C is more realistic for streaked shearwaters (40.5°C; Warham 1996). Since the subjects did not receive any food during the experiment, for the rCO_2_ calculation, we averaged the values of initial and final body water pools as the body water pool of each subject considering their body mass loss. We inferred the value of final isotope dilution space from the final body mass, assuming the same percentage of body water pool as measured for the initial body water pool.

One-pool model by Speakman (1997) (SP97):


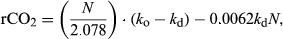


where 

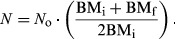


Two-pool model by Schoeller et al. (1986) (SCH86), as modified by Schoeller (1988):



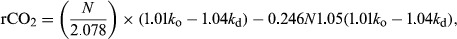


where 

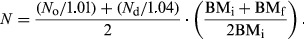


Two-pool model by Speakman et al. (1993) (SNG93):



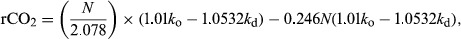


where 

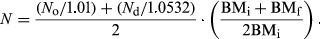


Two-pool model by Speakman (1993) (SP93):



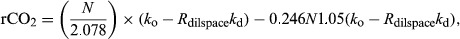


where 

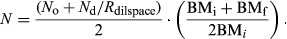


Two-pool model by Speakman (1997) (SP97):



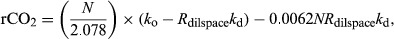


where 

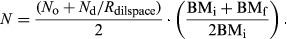


The water efflux (rH_2_O, mL/day) is equal to the sum of the water loss from respiration, skin, and excreta, and was computed using the elimination rate of hydrogen isotope from the equation of Bevan et al. (1995b) (based on Nagy and Costa 1980) as follows:



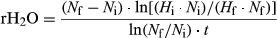


where 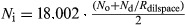, and 
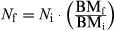
.

*H*_i_ (ppm) and *H*_f_ (ppm) are the initial and final enrichment of hydrogen isotope, respectively, and *t* (days) is the time between initial and final blood sampling being taken. To convert units in mL/day into energy equivalents, we assumed that 1 mL of CO_2_ = 25.11 J (RER = 0.8; Gessaman and Nagy 1988).

### Data analysis

To estimate metabolic rate via the DLW method, two or three replicate analyses are generally used to calculate the mean isotope values (Speakman 1997). These replicate analyses give an indication of the isotopic analytical variability (i.e., the magnitude of random error) in estimates of metabolic rate within individual (Speakman 1995). In our study, because the initial and final enrichments of oxygen and hydrogen isotopes were analyzed in duplicate, respectively, the number of potential combinations in calculated metabolic rate is 2^4^ = 16 estimates (two initial and two final samples in both oxygen and hydrogen isotopes) in each individual. The confidence intervals (95% CIs) and standard deviation of estimated metabolic rates were obtained using the 16 estimates. The coefficient of variation (%CV) was calculated by dividing the standard deviation by the metabolic rate calculated using the mean isotope values from two replicate analyses.

To compare the DLW method and respirometry, we used Passing–Bablok regression analysis to provide unbiased linear regression slopes and intercepts (Passing and Bablok 1983). Unlike ordinary least-squares regression, the Passing–Bablok regression analysis allows for imprecision in both the reference method (e.g., the respirometry) and the comparison method (e.g., the DLW method). There are two potential sources of systematic disagreement between methods of measurement: fixed and proportional error (Ludbrook 1997). For fixed error, one method gives values that are higher (or lower) than those from the other by a constant amount. For proportional error, one method gives values that are higher (or lower) than those from the other by an amount that is proportional to the level of the measured variable. Fixed error is indicated if the 95% confidence interval (CI) for the intercept of the Passing–Bablok regression does not include zero. Proportional error is indicated if the 95% CI for the slope of the Passing–Bablok regression differs from unity.

All data were analyzed using R version 3.0.1. (R Core Team 2013). We used *t*-tests (two tailed), ANOVA with Tukey’s HSD multiple comparison test, and Passing–Bablok regression analysis. Passing–Bablok regressions were run using the “mcreg” function in mcr package (Manuilova et al. 2015). Statistical analyses of %CV were performed after the data were arcsine transformed. We report our results without Bonferroni or similar adjustments on *P* values (see Rothman 1990; Perneger 1998). *P* values less than 0.05 were considered statistically significant. All values are presented as mean ± SD.

## Results

We recorded total energy expenditures measured by respirometry (TEE_resp_) ranging from 238.7 to 1137.0 kJ (see Table S1). TEE_resp_ in Groups B and C was on average 1.9 and 3.3 times higher than that in Group A (Table1). In Group B, there was statistically no difference between the TEE_resp_ within the first 24 h and second 24 h (*t*_*4*_ = 1.40, *P *=* *0.1). Mass-specific resting metabolic rates measured by respirometry (RMR_resp_) were significantly different among the groups (Table1). We found no statistical difference in RMR_resp_ measured on the ground in 2010 versus 2013 (*t*_*13*_ = 0.18, *P *=* *0.9), or in RMR_resp_ measured on the water in 2012 versus 2013 (*t*_*7*_ = 0.89, *P *=* *0.4). RMR_resp_ measured on the water (0.0519 ± 0.011 kJ·g^−1^·h^−1^; *n *=* *9) was to 3.4 times higher than RMR_resp_ on the ground (combined Group A and B: 0.0154 ± 0.0021 kJ·g^−1^·h^−1^; *n *=* *15).

**Table 1:** Metabolic rates, isotope turnover rates, and water efflux rates of streaked shearwaters on the ground or on the water. TEE_resp_ and RMR_resp_ represent total energy expenditure and resting metabolic rate measured using the respirometry, respectively. *k*_d_ and *k*_o_ represent the isotope turnover rate for ^2^H and ^18^O determined using the two-sample technique, respectively. Water efflux rate was calculated from hydrogen isotope turnover rate. Analyses among groups were performed by ANOVA with post hoc Tukey’s HSD multiple comparison test. Different superscripts identify means that differ significantly from each other (*P* < 0.05)

	Group A		Group B		Group C		Statistics	
	Mean	SD	Mean	SD	Mean	SD	*F*	*P*
Number of individuals	10		5		9			
Experimental condition	GROUND		GROUND		WATER			
Measurement duration (h)	24.4	0.2	48.1	0.1	24.3	0.3		
Initial body mass (g)	537	38	533	54	559	72	0.51	0.61
Final body mass (g)	503	39	485	50	539	75	1.70	0.21
TEE_resp_ (kJ)	258.6^A^	17.4	486.4^B^	98.4	860.9^C^	210.2	46.03	<0.001
RMR_resp_ (kJ·g^−1^·h^−1^)	0.0155^A^	0.0022	0.0153^A^	0.0021	0.0519^B^	0.0113	72.77	<0.001
Isotope turnover rate								
*k*_d_ (per day)	0.0639^A^	0.0564	0.0407^A^	0.0096	0.4248^B^	0.2257	18.43	<0.001
*k*_o_ (per day)	0.1218^A^	0.0500	0.0981^A^	0.0123	0.6527^B^	0.2703	28.25	<0.001
*k*_d_/*k*_o_	0.4536	0.2699	0.4101	0.0532	0.6192	0.0992	2.67	0.09
Water efflux rate (mL·day^−1^)	27.84^A^	15.81	27.91^A^	3.84	111.31^B^	48.66	19.36	<0.001

Initial isotope enrichments in Groups A, B, and C was 626.3 ppm (±41.5, *n *=* *10), 548.9 ppm (±43.8, *n *=* *5), and 554.5 ppm (±46.6, *n *=* *9) in ^2^H, respectively. In ^18^O, initial isotope enrichments in Groups A, B, and C was 2886.5 ppm (±57.3, *n *=* *10), 2870.1 ppm (±102.5, *n *=* *5), and 2861.0 ppm (±111.2, *n *=* *9), respectively. The depletion of ^2^H of body water from the initial enrichments was on average 6.1% (±5.1, *n *=* *10) in Group A, 7.8% (±1.9, *n *=* *5) in Group B, and 33.4% (±15.0, *n *=* *9) in Group C, respectively (see Table S2). Similarly, the depletion of ^18^O of body water from the initial enrichments was on average 11.5% (±4.3, *n *=* *10) in Group A, 17.8% (±2.0, *n *=* *5) in Group B, and 46.6% (±14.8, *n *=* *9) in Group C, respectively (see Table S2). The elimination rate of hydrogen (*k*_d_) and oxygen isotope (*k*_o_) in Group C was significantly higher than all other conditions, but the *k*_d_/*k*_o_ ratios did not differ among the groups (Table1). The water efflux rate in Group C was also significantly higher than in all other conditions (Table1).

TEE_resp_ along with corresponding total energy expenditures measured by the DLW method (TEE_dlw_) and the ratio between the two estimates are detailed in Table2 (see Table S3 for individual values). Regardless of the equations, mean value of TEE_dlw_ in Group A matched with TEE_resp_ (Table2). In Groups B and C, the equation by Speakman et al. (1993; two-pool model) provided the most accurate TEE_dlw_, whereas the equation of Speakman (1997; one-pool model) provided the least accurate results (Table2). Mean TEE_resp_ corresponded to 104%, 81%, and 76% of mean TEE_dlw_ calculated by the equation by Speakman et al. (1993) in Groups A, B, and C, respectively (Table2).

**Table 2:** Comparison of total energy expenditures measured by the DLW method (TEE_dlw_) and respirometry (TEE_resp_) in streaked shearwaters. TEE_dlw_ were calculated from the five equations from the foregoing studies below. Estimate and ratio represent TEE_dlw_ value calculated using each equation and the ratio between TEE_dlw_ and TEE_resp_, respectively

Equations*	Group A		Group B		Group C	
	Mean	SD	Mean	SD	Mean	SD
Experimental condition	Ground		Ground		Water	
Measurement duration (h)	24.4	0.2	48.1	0.1	24.3	0.3
TEE_resp_ (kJ)	258.6	17.4	486.4	98.4	860.9	210.2
TEE_dlw_ (kJ)						
SNG93 (T)						
Estimate	248.3	113.2	602.9	86.4	1128.4	316.3
Ratio	0.952	0.398	1.267	0.216	1.308	0.212
SP93 (T)						
Estimate	253.4	111.9	612.1	87.8	1167.3	329.4
Ratio	0.971	0.392	1.286	0.217	1.351	0.216
SCH86 (T)						
Estimate	252.7	112.0	610.9	87.6	1162.4	327.7
Ratio	0.969	0.393	1.283	0.217	1.346	0.215
SP97 (T)						
Estimate	264.0	120.0	640.7	91.8	1201.3	337.0
Ratio	1.012	0.421	1.346	0.229	1.392	0.225
SP97 (O)						
Estimate	271.4	112.5	645.6	94.4	1279.1	367.2
Ratio	1.041	0.393	1.355	0.227	1.477	0.230

* Five equations were used to calculate metabolic rate: SCH86, equation 6 from Schoeller et al. (1986); SP93, equation 4 from Speakman (1993); SNG93, equation 3 from Speakman et al. (1993); SP97, equation 7.17 and 7.43 from Speakman (1997). T and O in parentheses indicate the two- and one-pool model, respectively.

**Figure 2:**
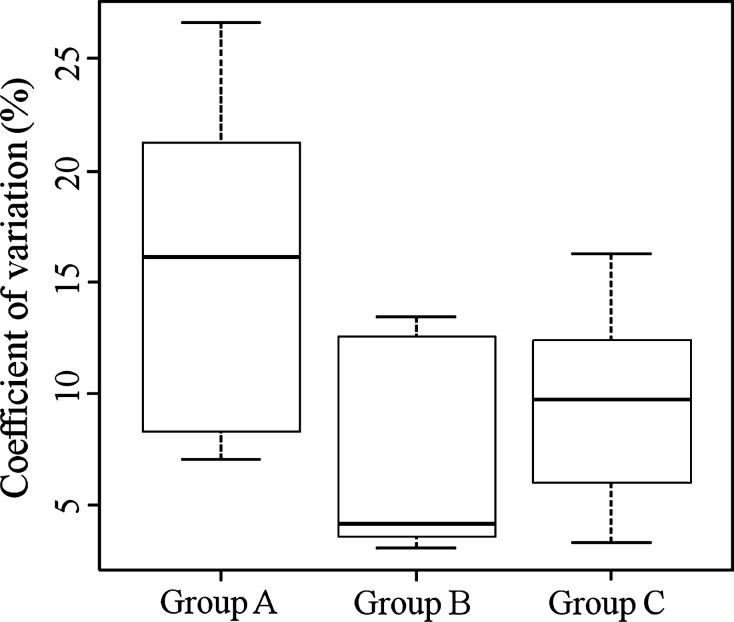
Effect of isotopic analytical variability in total energy expenditures measured by the doubly labeled water method in streaked shearwater by three different experimental conditions: at rest on the ground for 24 h (Group A; *n *=* *10) or for 48 h (Group B; *n *=* *5), and at rest on the water for 24 h (Group C; *n *=* *9). Duplicate isotopic analyses of serum samples provided 16 calculated metabolic rates in each individual. The coefficient of variance of the 16 metabolic rates in each individual gives an indication of the isotopic analytical variability on metabolic rates measured by the doubly labeled water method. The coefficient of variation calculated in Group B was significantly lower than that in Group A (*P *=* *0.037). Similarly, the coefficient of variation in Group C tend to be lower than that in Group A (*P *=* *0.0503).

For all calculations of precision and accuracy for the DLW method, we used the results from the two-pool model of Speakman et al. (1993) (see Table S4). The % CVs (relative impacts of isotopic analytical variability in energy expenditure) of TEE_dlw_ in Group B were significantly lower than with those in Group A (*t*_13_ = 2.47, *P *=* *0.037; Fig.2). The %CVs in Group C also tended to be lower than those in Group A, but the difference was statistically insignificant (*t*_17_ = 2.12, *P *=* *0.0503; Fig.2).

**Figure 3:**
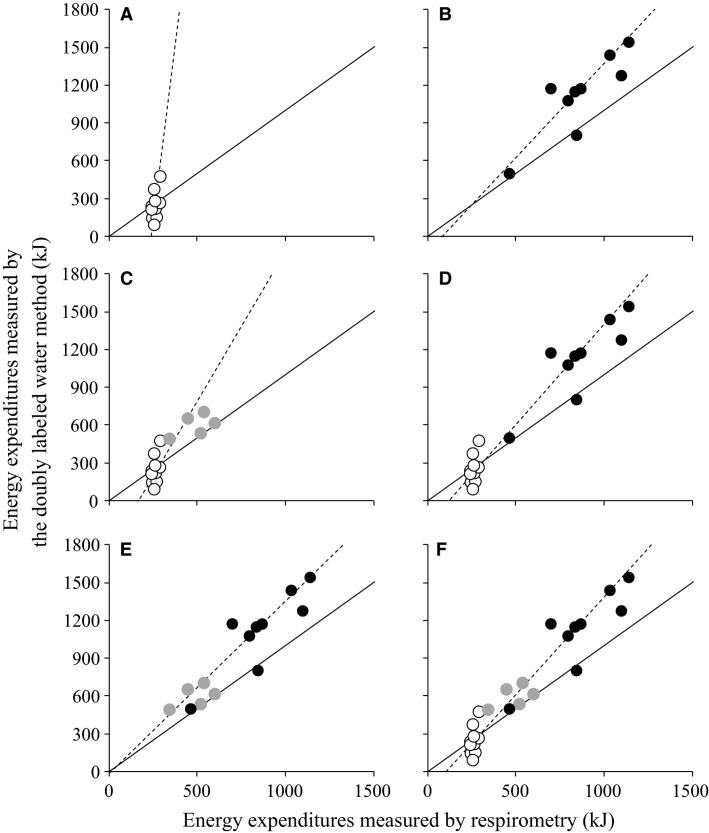
Passing–Bablok regression derived from total energy expenditure in streaked shearwater measured by the doubly labeled water (DLW) method and respirometry using individuals of Groups A, B, and C (see Materials and Methods section). (A) Using Group A; (B) using Group C; (C) using Groups A and B; (D) using Groups A and C; (E) using Groups B and C; (F) using Groups A, B, and C. White, gray, and black circles represent the resulting estimates of Groups A, B, and C, respectively. The black dashed line and solid line indicates the Passing–Bablok regression line and the line of identity, respectively.

To test the linearity between the DLW method and respirometry, six combinations of groups (A, C, A–B, A–C, B–C, and A–B–C) were tested for agreement between the DLW method and respirometry by Passing–Bablok regression analysis, except for Group B only because of small sample size. Among all tests, the combination of Group B and C is the closest model to identical (Fig.3, Table3). The coefficient of determination (*R*^2^) of the best model is 0.82. Although the best model showed the intercept value with insignificant fixed error, the slope value was significantly overestimated and differed from unity (Table3).

**Table 3:** Verifications by Passing–Bablok regression analysis for fixed error and proportional error against the respirometry criterion in streaked shearwaters

No.	Dataset*	Intercept	95% CI	Fixed	Slope	95% CI	Proportional	Coefficient of determination
			for intercept**	error**		for slope**	error**	
1	Groups B + C	−14.12	−362.77 to 172.84	Nonexistence	1.37	1.04 to 1.79	Existence	0.82
2	Group C	−119.48	−985.99 to 486.82	Nonexistence	1.50	0.80 to 2.50	Nonexistence	0.65
3	Groups A + B + C	−130.28	−311.49 to −36.67	Existence	1.52	1.32 to 1.81	Existence	0.91
4	Groups A + C	−195.19	−343.81 to −86.63	Existence	1.60	1.37 to 1.87	Existence	0.91
5	Groups A + B	−398.47	−1274.59 to −120.16	Existence	2.38	1.41 to 5.41	Existence	0.27
6***	Group A	−2650.18	N/A	N/A	11.17	N/A	N/A	N/A

* Subjects in three groups were exposed with different experimental conditions: at rest on the ground for 24 h (Group A) or for 48 h (Group B), and at rest on the water for 24 h (Group C).

** Values are the 95% CI for fixed error (intercept ≠ 0) and proportional error (slope ≠ 1) for each method against the respirometry criterion.

*** Passing–Bablok regression analysis gave false values on 95% CI for intercept and slope and could not evaluate the fix and proportional error.

## Discussion

The aim of this study was to investigate whether the amount of eliminated isotopes affects the reliability of total energy expenditures measured by the DLW method (TEE_dlw_) in streaked shearwaters. For this purpose, we designed different experimental conditions with respirometric chambers: at rest on the ground for 24 h (Group A) or for 48 h (Group B), and at rest on the water for 24 h (Group C). We used two indices to measure the reliability of the DLW method: precision (i.e., the closeness of repeated measurements within an individual) and accuracy (i.e., the closeness of an estimated value to respirometry). The mean resting metabolic rates measured by respirometry (RMR_resp_) on the ground (0.0154 kJ·g^−1^·h^−1^) was equal to the predicted RMR (0.0155 kJ·g^−1^·h^−1^) that was calculated (using body mass = 500 g) from equation 11.6 by Ellis and Gabrielsen (2002). Similar to seabirds such as cormorant, shag, and penguin, which show RMRs on the water to be over three times higher than RMR on the ground (reviewed in Richman and Lovvorn 2011), we found that sitting in water increased RMR_resp_ in streaked shearwaters by a factor of 3.4 and also increased the elimination rates of hydrogen and oxygen isotopes (*k*_d_, *k*_o_) and water efflux rate. With regard to the accuracy of the DLW method, mean TEE_dlw_ matched mean total energy expenditures measured by respirometry (TEE_resp_) in Group A, whereas TEE_dlw_ was overestimated in Groups B and C compared with TEE_resp_. The TEE_dlw_ in Groups B and C, however, showed higher precision (i.e., less random error) for the DLW method than the TEE_dlw_ estimates derived in Group A. Although the best regression model (No. 1 in Table3) detected systematic (proportional) error between TEE_dlw_ and TEE_resp_, the analysis also showed a high correlation between the methods. Our study clearly demonstrates that differences in experimental design influence both the precision and accuracy of the DLW method through random and systematic errors.

### Differences in precision depending on experimental conditions

Compared with Group A, TEE_dlw_ in Groups B and C not only had lower isotopic analytical variability (i.e., random error) (Fig.2), but also had lower accuracy (see below). This indicates that relative high isotope elimination alleviates the effects of analytical variability of IRMS in TEE_dlw_ and improves the precision of the DLW method, as found by Sparling et al. (2008). Nagy (1980) and Gales (1989) recommended that the final oxygen isotope enrichment above background should be lower than approximately 50% of the initial enrichment above background to reduce the effects of errors in isotopic analysis, as supported by our result from Group C. Almost all previous DLW validation studies have not counted or described the extent of eliminated isotopes, so the degree to which their results are influenced by the random error in isotopic analysis is not known.

Presumably because the impact of analytical variability on TEE_dlw_ was reduced in Groups B (longer experiment period) and C (measured on water), the best regression model (Groups B and C, No. 1 in Table3) showed a high correlation between TEE_dlw_ and TEE_resp_. This result is consistent with a previous simulation study of DLW method precision, which concluded that interindividual comparisons in relation to ecological variables by the method are robust (Peterson et al. 1998). Several validation studies have advised caution when applying the DLW method in individual-based measurements because individual estimates from the DLW method can differ by more than 40% from those derived from the respirometry method (Bevan et al. 1995b; Boyd et al. 1995; Jones et al. 2009). The design and species used for these validations (turtle and water bird), however, have some disadvantages for applying the DLW method: relative short experimental period (Boyd et al. 1995) and high water efflux relative to CO_2_ production (i.e., *k*_d_/*k*_o_ ratio of 0.8 and above) and/or low metabolic rate (Bevan et al. 1995b; Jones et al. 2009). High water efflux relative to CO_2_ production creates a situation where the difference in isotope turnover of hydrogen and oxygen is small (Jones et al. 2009). Thus, errors in isotopic analysis can easily influence calculated metabolic rates (see Fig. 4 of Nagy 1980). In our study, streaked shearwaters have different physiologies in that they are a homoeothermic with low *k*_d_/*k*_o_ ratios (mean ratios: 0.454 in Group A, 0.410 in Group B, and 0.619 in Group C). Thus, although a correction for systematic error is necessary for a valid estimation (see below), our results suggest that a high extent of isotope elimination provides DLW estimates with high precision, which may reflect relative individual estimates.

### Differences in accuracy depending on experimental conditions

In agreement with many other validation studies of the DLW method (reviewed in Speakman 1997, 1998), the mean TEE_dlw_ across the group of birds in Group A was close to the mean TEE_resp_ (Table2), whereas TEE_dlw_ in Groups B and C were highly overestimated by proportional error (Tables2 and 3). The differences in accuracy depending on experimental conditions may be produced by the relative difference between random (e.g., impact of isotopic analytical variability on TEE_dlw_) and systematic error (e.g., degree of mismatch between the assumptions of the DLW method and physiological conditions in streaked shearwater). Since random error within a group generally cancels itself out, it has little influence on the average of the group (Taylor 1997). Because the TEE_dlw_ in Group A includes relatively large isotopic analytical effect (Fig.2), the match between mean TEE_dlw_ and TEE_resp_ in Group A is consistent with the characteristics of random error. On the other hand, the TEE_dlw_ in Groups B and C eliminated the random error by higher extent of isotope eliminations (see above), so the estimates may show systematic error. When the DLW method is used in the field, the situation (e.g., the extent of eliminated isotopes) more closely resembles conditions in Groups B and C compared to Group A. Thus, the correction using the best regression model (TEE_dlw_ = 1.37 TEE_resp_ − 14.12; Table3) may help to provide actual TEE of streaked shearwater in the field. The correction may be effective before three half-lives of oxygen isotope (i.e., one-eighth of initial concentration; Nagy 1983), because the elimination rate of oxygen isotope may change if the concentrations of the oxygen isotope at final sampling are too close to the concentrations at background.

What is the cause of the systematic error in streaked shearwater? The DLW method relies on distinguishing the elimination curves of oxygen and hydrogen isotopes (Fig.1). Thus, the overestimated TEE_dlw_ depends on the rates of isotope eliminations and suggests that the *k*_d_ was underestimated, the *k*_o_ was overestimated, or both processes occurred simultaneously. The fact that our best regression model showed proportional error suggests that the estimated isotope elimination rates constantly stray outside of the range of actual rates. With regard to *k*_d_, the mean water efflux rate in individuals of Groups A and B, which was measured by the *k*_d_, was 32% above the level (21.2 mL·day^−1^) predicted for birds based on the allometric equation with phylogenic analysis (Williams 1996). Thus, for birds in both Groups A and B, *k*_d_ is unlikely to be underestimated. In Group C, water efflux rate increased 4.0 times compared with those in Groups A and B, and the increment exceeded the level of the increment in metabolic rate (3.4 times). Therefore, although there is little information about water efflux rate in birds while floating on the water, this result suggested that for birds in Group C, we did not underestimate *k*_d_. So, the overestimated TEE_dlw_ in both Groups B and C may be caused by the overestimation of *k*_o_. As the cause of the overestimation in *k*_o_, previous studies pointed out the possibilities of additional irreversible loss of oxygen isotope to urea through the ornithine–arginine cycle (Sparling et al. 2008) or to ketone bodies (Guidotti et al. 2013). Although both the previous and present studies have not isolated the specific cause of the *k*_o_ overestimation, the cause of the systematic error may be partially due to additional substances by fasting condition in our experiments, which increase the production of ketone bodies such as *β*-hydroxybutyrate (Totzke et al. 1999).

As the other explanations for the systematic error of the DLW method, total body water pool (TBW) might have caused the overestimated TEE_dlw_. Since TBW estimated by isotope dilution method was used to calculate the TEE_dlw_ (see Materials and Methods section), the error of TBW estimation would reduce the accuracy of the DLW method. However, previous studies suggest that TBW estimated by the isotope dilution method matched actual TBW in seabirds (accuracy: −4.8 to +7.0%; Jacobs et al. 2012). Thus, although we have no actual TBW values for streaked shearwaters, the impact of TBW on the overestimated TEE_dlw_ should be limited.

Our results suggest that the DLW method accurately estimates the mean metabolic rate of animals only in some circumstances (Speakman 1998; Butler et al. 2004). The DLW method, thus, should be used with caution especially when characterizing interspecies difference of FMR. Most of previous validation studies in birds, reptiles, and mammals have been conducted under only one experimental condition (i.e., fixed measurement period and metabolic rate of subjects) within a study (reviewed in Speakman 1997). Further validation study is required to evaluate accuracy of the DLW method and to understand factors affecting the accuracy for a larger range of species.

## Conclusion

Our results indicate that the precision of the DLW method improves substantially in experiments that more closely resemble field conditions, that is, longer sampling intervals or higher metabolic rate. This effect is primarily mediated by reduced isotopic analytical variability in the energy expenditure estimates produced by the DLW method. In these conditions, we found a high correlation between the total energy expenditure estimates derived by the DLW method and respirometry. The result in Group A, in contrast (short sampling interval and lower metabolic rate) showed considerable variation in TEE_dlw_ compared to little variation in TEE_resp_, so the two measurements correlated poorly. The results from Group A were consistent with findings from several similar earlier DLW studies, which were inadequate for individual-based measurements in free-ranging animals (e.g., Bevan et al. 1995b; Jones et al. 2009). Certain criteria, however (i.e., low water efflux relative to CO_2_ production, and large isotope elimination), as satisfied by Groups B and C in our study, may evidently enable adequate individual-based measurements of energy expenditure using the DLW method. Our results also support Shaffer’s (2011) suggestions that individual DLW estimates partially contributes to a relative index of individual effort in free-ranging animals. However, with an overestimation of TEE_dlw_ of greater than 30% in situations with high isotope elimination implies that, although the DLW method provides good correlations between energy expenditure and ecological or behavioral variables within species, the method does not always provide accurate differences in energy expenditure between species. Since few validation studies have discussed the actual precision and accuracy of the DLW method for field use, our study emphasizes the need for further validation studies for the refinements and revisions of the usage of the DLW method in the field. Nevertheless, the recent dramatic increase in studies of alternative behavioral and resource allocation strategies has been fettered by the lack of a suitable method for quantifying individual differences in energy expenditure in free-living animals and our study indicate that the DLW method can perform adequately for such aims.

## Supporting Information

**Table S1.** Details of all experimental trails. TEE_resp_ is total energy expenditure as measured by respirometry. **Table S2.** The details of isotope turnover rates (hydrogen, *k*_d_ and oxygen, *k*_o_), dilution spaces (hydrogen, *N*_d_ and oxygen, *N*_o_) and water efflux rate. *k*_d_/*k*_o_ and *N*_d_/*N*_o_ are the ratios of the turnover and dilution space of the two isotope labels, respectively. Water efflux rate was calculated from hydrogen isotope turnover rate. **Table S3.** Comparison of total energy expenditure measured by the DLW method (TEE_dlw_) and respirometry (TEE_resp_). Estimate and ratio represent TEE_dlw_ value calculated using each equation and the ratio between TEE_dlw_ and TEE_resp_, respectively. **Table S4.** Results of analytical variability on total energy expenditure derived from the DLW method (TEE_dlw_). Upper and lower 95% confidence limits, standard deviation and %CV of TEE_dlw_ were calculated using each individual oxygen and hydrogen isotopes analyzed in duplicate (detailed in Materials and Methods section).
